# Elementary Treatise on Diseases of the Skin, for the Use of Students and Practitioners

**Published:** 1877-05

**Authors:** 


AN ELEMENTARY TREATISE ON DISEASES OF THE SKIN, for the use of Students and Practitioners. By Henry G. Piffard, M.D., Professor of Dermatology, University of the City of New York; Surgeon to the Charity Hospital, to the New York Dispensary for Diseases of the Skin, etc.; with illustrations. McMillan & Co., London and New York. 1876.

We have scanned over this work of 375 pages, and are very much pleased with it. The author, after giving a very comprehensive description of the anatomy, physiology, and pathology of the skin, together with a chapter each on symptomatology and diagnosis, enters into a comparison of the various plans of classification of skin diseases. From these—comprising the *lesional* of PLEUCK; the *natural* of ALIBERT, and the *pathological* of HEBRA—the author has preferred that of ALIBERT with modifications, and upon the basis of the etiological or natural relationship of skin diseases, has founded his classification. Upon mature deliberation and extended experience, the following general plan has been elaborated:

I. Diathetic affections.
II. General non-diathetic affections.
III. Reflex affections.
IV. Local affections.
V. Affections of uncertain nature.

The elaborate details, and the illustrations, in connection with the distinct and comprehensive classification, render the work very valuable and acceptable. Students will find in it a treatise easily understood, and practitioners will have much of that obscurity dispelled which the intricate and labored works of old have created. As a book of easy reference, and other conspicuous merit, we readily commend it to the profession.

